# Septic Portal Vein Thrombosis, Clinical Presentation, and Management

**DOI:** 10.7759/cureus.19840

**Published:** 2021-11-23

**Authors:** Simran Anand, Chukwuemeka A Umeh, Curren Giberson, Elias Wassel, Anphong Nguyen, Hayden Porter, Prithi Choday, Harpreet Kaur, Ankur Kundu, Jose Penaherrera

**Affiliations:** 1 Internal Medicine, St. George's University School of Medicine, True Blue, GRD; 2 Internal Medicine, Hemet Global Medical Center, Hemet, USA; 3 Radiology, Hemet Global Medical Center, Hemet, USA

**Keywords:** hypercoagulopathy, superior mesenteric vein thrombosis, hepatic abscess, septic portal vein thrombosis, pylephlebitis

## Abstract

Pylephlebitis, otherwise known as septic portal vein thrombosis, is an infective suppurative thrombosis of the portal vein and/or its intra-hepatic branches. It is a diagnosis that is frequently missed but easily treated with antibiotics. Therefore, it should be considered early on in any patient presenting with fever, abdominal pain, leukocytosis, and evidence of portal vein thrombosis on a CT scan. In this case report, we discuss a case of pylephlebitis as well as the etiologies, diagnosis, and treatment of septic portal vein thrombosis.

## Introduction

Pylephlebitis, otherwise known as septic portal vein thrombosis, is an infective suppurative thrombosis of the portal vein and/or its intra-hepatic branches. The most common etiology of pylephlebitis is an intra-abdominal infection that drains into the portal vein. As this diagnosis is extremely rare [1], its detection is frequently delayed and carries a high morbidity and mortality rate [2]. Furthermore, pylephlebitis is a diagnosis that is easily treated with early antibiotics and anticoagulation, therefore, it is imperative to consider in the differential diagnosis of or as a complication of intra-abdominal infection. This case report will present a case of pylephlebitis and discuss the different etiologies and treatments of septic portal vein thrombosis.

## Case presentation

A 24-year-old male with a history of gastritis presented to the emergency department with a chief complaint of epigastric abdominal pain. Of note, the patient had presented to the emergency department (ED) 10 days prior with the same complaint. At that time, point-of-care labs showed only mild hypokalemia. The patient did not have any imaging done and was discharged after tramadol and omeprazole improved his pain. During the second visit, the patient reported similar pain, this time worse and associated with subjective fever and chills. Upon further questioning, he also endorsed nausea, non-bloody and non-bilious emesis, and diarrhea over the past two weeks. He reported about two to three episodes of non-bloody diarrhea per day.

The patient’s past medical history is significant only for gastritis and three pack-years of tobacco use. His medication history included only omeprazole. The patient denied having a personal or family history of hypercoagulability, thromboses, or cancer. He denied any alcohol or illicit drug use. He recently immigrated from Mexico and endorsed eating street food and drinking tap water in Mexico prior to this presentation.

On arrival, the patient’s vital signs were as follows: temperature 98.3°F, respiratory rate 18 breaths/minute, blood pressure 116/73 mmHg, heart rate 83 breaths per minute (BPM), and oxygen saturation 96% on room air. His examination was notable for only mild epigastric and right upper quadrant tenderness to deep palpation. The abdomen was soft and non-distended without peritoneal signs. There was no hepatomegaly or evidence of jaundice.

Labs were significant for a white blood cell count of 21,300/mm^3^ with significant neutrophilia. There was no anemia or thrombocytopenia. The international normalized ratio (INR) was 1.5. The patient’s liver enzymes, aspartate transaminase (AST), and alanine transaminase (ALT) were within normal limits, while alkaline phosphatase was significantly elevated at 197 IU/L (reference range: 34-104). Lipase was normal. Total bilirubin and direct bilirubin were slightly elevated at 1.1 umol/L (reference range: 0.3-1.1) and 0.5 umol/L (reference range: 0.1-0.3), respectively.

**Figure 1:**
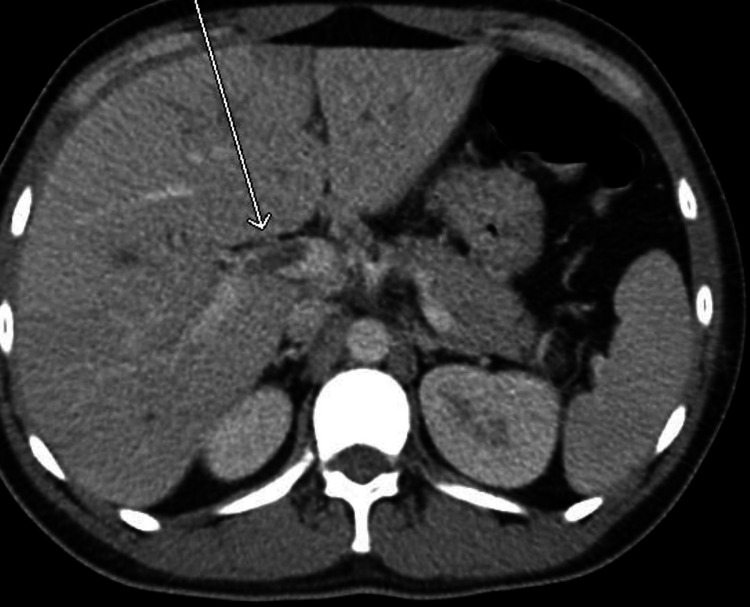
CT abdomen (axial view) showing portal vein thrombosis

**Figure 2:**
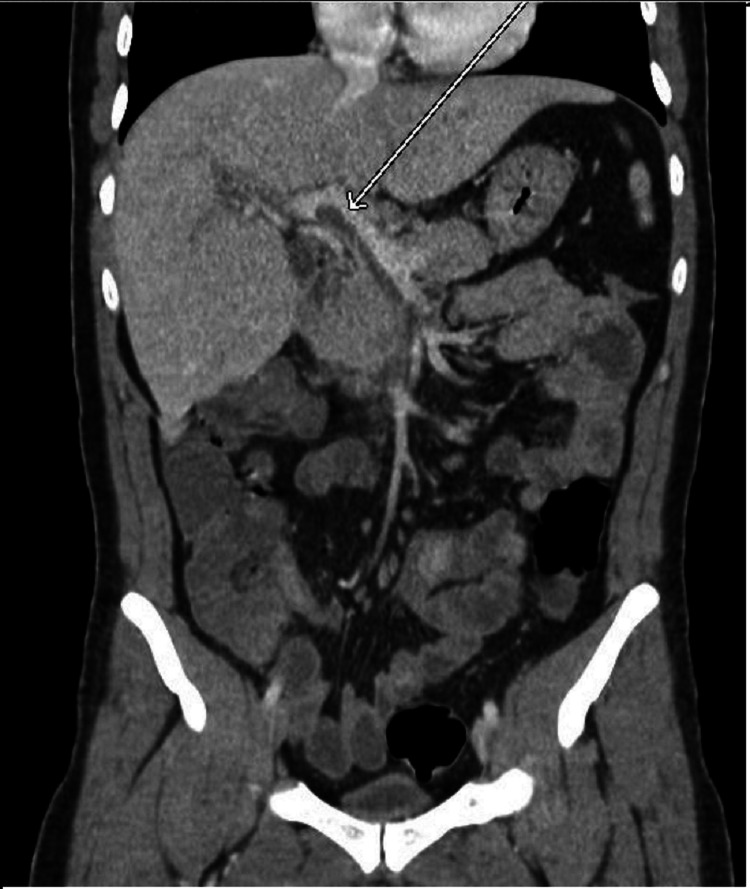
CT abdomen (coronal view) showing portal vein thrombosis

**Figure 3:**
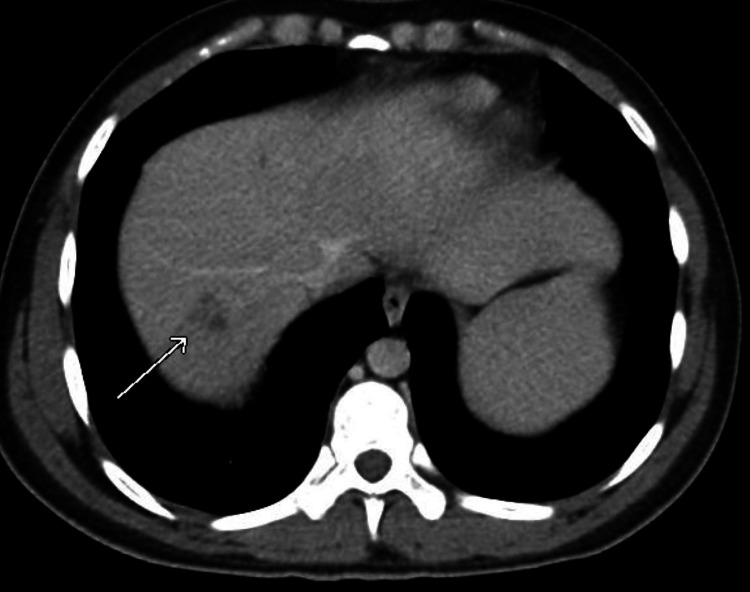
CT abdomen (axial view) showing possible liver abscess

**Figure 4:**
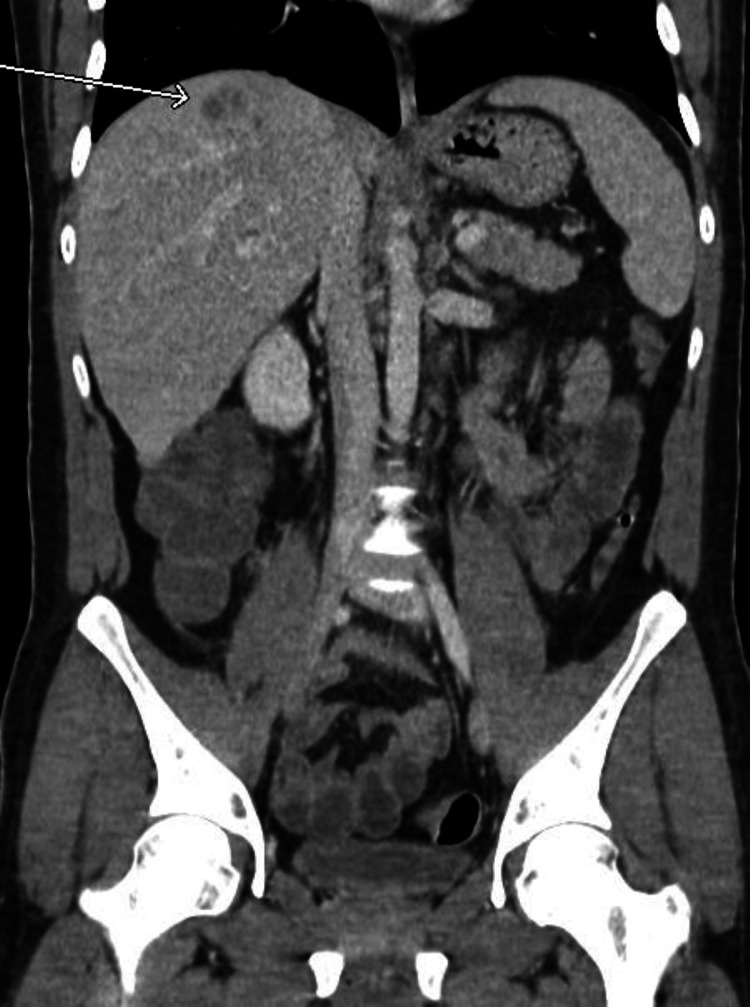
CT abdomen (coronal view) showing possible liver abscess

Since this was the patient’s second visit to the ED with similar epigastric abdominal pain, a computed tomography (CT) abdomen/pelvis with intravenous (no oral) contrast was ordered by the emergency room physician. The scan demonstrated a thrombus within the portal and superior mesenteric vein (Figures 1, 2). There was also an ill-defined collection of rounded subcentimeter hypodensities in the right hepatic lobe of the liver approximately 1.7 cm in size; differential diagnosis included intrahepatic portal vein thrombi versus abscess (Figures 3, 4). The portal vein was mildly dilated at 1.8 cm. There was possible evidence of gastroenteritis due to multiple non-dilated fluid-filled loops of the bowel. This CT scan showed no evidence of appendicitis. An ultrasound of the gallbladder and liver was also performed, showing mild hepatomegaly without any evidence of focal hepatic lesions. A hypercoagulability workup including protein C/S, antithrombin III, and factor V Leiden was also collected and was within normal limits other than a slightly low protein C activity of 54% (reference range: 73-180). This may be due to a congenital deficiency or due to an acute clot burden. The patient was advised to follow-up on this finding outpatient.

On arrival, the patient was non-toxic and did not meet any systemic inflammatory response syndrome (SIRS) criteria other than leukocytosis. Because of this, no blood cultures were collected during his initial presentation. However, on day three of his hospital stay, the patient developed a fever of 101.5°F and became tachycardic to 128 beats/minute. Acetaminophen was administered with a resolution of the fever. Blood cultures were collected at that time, notably after the initiation of Flagyl therapy. Two sets of blood cultures were negative, potentially because they were collected after antibiotic commencement.

The patient was started on Lovenox 75 mg subcutaneously every 12 hours (weight-based dosing: 1 mg/kg) for his portal vein thrombosis. He was also started on intravenous Flagyl 500 mg every eight hours and ceftriaxone 2 g daily for pylephlebitis. Infectious disease was consulted and recommended that the patient continue IV antibiotics for two weeks due to potential hepatic abscess leading to septic portal vein thrombosis. A tagged WBC scan on day five of the patient’s admission (ordered by infectious disease) showed no nuclear evidence of focal hepatic lesions such as abscess, however, this WBC scan was ordered after the initiation of antibiotics. Infectious disease recommended continuation of oral antibiotics for six weeks post-discharge.

The patient improved rapidly with IV antibiotics and anticoagulation. He was without abdominal pain, fevers, nausea, vomiting, and diarrhea after day three of admission. A repeat CT abdomen pelvis with oral contrast was performed two weeks after the patient’s initial admission and showed persistence of the portal vein thrombus, notably with an improved clot burden and decreased dilation of the portal vein from 18 mm to 14 mm. The repeat CT scan also demonstrated a new finding, early acute appendicitis. There was a blind-ending tubular fluid-filled appendix measuring 12 mm in diameter (compared to the previous CT’s 5 mm appendix) with mild adjacent inflammatory changes and right lower quadrant mesenteric lymphadenopathy. Though we were meant to discharge the patient after the repeat CT scan, we thought it would be prudent to obtain a surgical consult as this infection may have been the initial source of the septic portal vein thrombus. Our surgical consult did not think appendectomy was necessary as the patient remained asymptomatic, there was no evidence of luminal obstruction by appendicolith, and there would be a risk for thrombosis in the post-operative period if anticoagulation was stopped. They recommended continued medical management with strict return instructions. The patient himself also was eager to be discharged from the hospital and declined an appendectomy at that time. He was discharged with a six-week course of oral Flagyl and Levaquin as well as anticoagulation with Xarelto. Because the thrombus extended into the patient’s superior mesenteric vein, we recommended he continue Xarelto for three to six months post-discharge.

## Discussion

Pylephlebitis can complicate some intra-abdominal infections, most notably appendicitis [3]. However, septic portal vein thrombosis has been noted to complicate courses of diverticulitis, cholangitis, inflammatory bowel disease, pancreatitis, and even transrectal prostate biopsy. A source of infection was found in 68% of cases in a report done in 1995 [4]. The diagnosis was 100% fatal in 20 cases reported in 1948, and mortality is still significant at 11-32% in the modern-day era [2,4,5].

Pylephlebitis extends to the superior mesenteric vein in 42% of cases and is associated with a pyogenic liver abscess in 37% of cases. Most cases that involve hepatic abscesses also include a contiguous intra-abdominal infection such as diverticulitis or pancreatitis [6-9]. Patients with pyogenic liver abscesses often have minimal clinical features and may present with a waxing and waning course over a few weeks [10]. Jaundice, elevated AST/ALT, and elevated bilirubin are infrequent findings, however, alkaline phosphatase (ALP) elevation has been seen in most patients, especially those with a contiguous pyogenic hepatic abscess [11].

Bacteremia is present in 88% of cases of pylephlebitis and is considered a significant predisposing factor [4]. The most common infections are polymicrobial and involve *Bacteroides fragilis*, *Escherichia coli*, and *Klebsiella pneumoniae*. Blood cultures should be obtained prior to administering antibiotics, however, negative blood cultures should not preclude the diagnosis.

A clinical diagnosis of pylephlebitis can be made when a patient presents with fever, abdominal pain, leukocytosis, and portal vein thrombosis. Fever was found in 100% of cases in a review of 19 cases of pylephlebitis [4] and 87% of patients in another study of 67 patients [9]. Radiological diagnosis of pylephlebitis can be made with CT scan, ultrasound, magnetic resonance imaging (MRI), and fluorodeoxyglucose-positron emission tomography (FDG-PET). The diagnostic imaging study of choice is the CT scan, as it is most likely to reveal a contiguous source of infection from the abdomen. As pylephlebitis is a rare diagnosis, the sensitivity and specificity of these tests are not yet known. Follow-up imaging is recommended about five to seven days after initiation of antibiotic therapy to identify thrombus extension or any other complications [12].

As pylephlebitis is a highly uncommon diagnosis, the use of both antibiotic and anticoagulative therapy is still debated, with no randomized controlled studies to guide treatment. Some authors recommend antibiotics for about four to six weeks and state that antibiotics should be given parenterally until there is a significant clinical response (usually two to three weeks). Antibiotics of choice include combination therapy with metronidazole plus either ceftriaxone, cefotaxime, ciprofloxacin, or levofloxacin or monotherapy with Zosyn, Unasyn, or a carbapenem.

Anticoagulation for pylephlebitis is a currently debated topic with no published guidelines to guide clinical decision-making. Two studies demonstrate decreased mortality in patients who were given anticoagulation [12,13], and one study shows an increased rate of thrombus resolution [14]. The duration of anticoagulation is also debated; some authors currently recommend treating until the patient has clinically improved or the thrombus stabilizes. The mean time for this was found to be 4.5 months [14]. Anticoagulation is more likely to benefit patients with worsening clinical symptoms such as continued bacteremia or fever. It is also more likely to be beneficial in cases of thrombosis progression into the mesenteric veins, notable ischemia, or when patients have a positive hypercoagulability workup. Anticoagulation also lessens the likelihood of chronic portal vein thrombosis and resulting portal hypertension via recanalization of the portal vein [9]. Direct oral anticoagulants (DOACs) have been used successfully for anticoagulation [9,14].

Other treatments for pylephlebitis include thrombolytic therapy, aspiration of thrombus, or surgical removal. These therapies are not currently recommended due to lack of experience and efficacy in performance and potential recurrence of thrombus. These therapies should be considered only in patients who do not respond well to antibiotics or anticoagulation.

## Conclusions

Pylephlebitis or septic thrombosis of the portal vein is a rare diagnosis that should be considered in patients presenting with fever, abdominal pain, bacteremia, and evidence of portal vein thrombosis on imaging studies. The most common etiologies include intra-abdominal infections that spread into the portal vein. Treatment includes antibiotic therapy for several weeks, and the use of anticoagulation is still debated.
